# Annotations

**Published:** 1904-04-02

**Authors:** 


### ANNOTATIONS.

### Cornwall as a Health Resort.

THE old-world land of "Tre, Pol and Pen" has long been a much-loved region of delight to artist, archæologist and naturalist, but as a country rich in resorts peculiarly suited to the needs of the delicate and the convalescent as well as the sufferer from lung and other affections Cornwall has been sadly neglected. Englishmen have been attracted far afield to much-lauded and much-advertised foreign stations and have been forgetful of the admirable sanatorium which awaits development in the near west of the homeland. At last railway enterprise seems likely to bring the advantages of Cornwall as a place where health may be restored and physical and mental energy renewed before the attention of both medical and lay public. We have before us a little book entitled *The Cornish Riviera* published by the Great Western Railway Company, in which is set forth the facilities which have recently been provided to render the pleasantest localities "in the Delectable Dutchy" easily accessible to visitors all the year round. Experience has already clearly demonstrated that in this Western region of tradition and romance, with its rugged coastline and rolling sea, conditions prevail which are peculiarly suited to the wants of those seeking health and pleasure. Cornwall might well be claimed as an all-the-year-round health resort. Its advantages as a winter station need to be more clearly recognised: Much sunshine prevails, the temperature is equable, and the air peculiarly soft and yet invigorating. The land of Lyonesse may well become as the Rev. S. Baring Gould prophesies, "The Sanatorium of the Future." Excellent arrangements for comfortable travelling are now provided and the Great Western Railway Company are very wisely opening up certain districts peculiarly suited for development as desirable health stations by means of a well-equipped service of motor-cars. We commend *The Cornish Riviera* to all in search of an ideal health and pleasure resort.

### The Treatment of Habitual Inebriates.

THE first report of the Inspector for Scotland under the Inebriates Act has just been issued and, containing as it does the experiences of a skilled medical observer, Dr. J. C. Dunlop, it is a document of some importance. The total number of cases brought under review is 92 and it is so far satisfactory to note that regarding the subsequent conduct of those who have been discharged after a period of treatment fully 50 per cent. are claimed either as cured (on the basis of 12 months' abstinence) or as "hopeful cases," the latter term being applied to those who have remained abstinent since discharged but who have not yet obtained the 12 months' guarantee. As was to be expected beforehand, the length of the period of discipline is an important element in reference to the success or failure of the treatment. Thus of 23 persons discharged after a course of less than six months no fewer than 15 relapsed, while of 20 who remained for periods ranging from 6 to 12 months only 4 relapsed. Another point of interest in reference to treatment is that in none of the institutions is reliance placed in any form of drug treatment. Suitable work and recreation, exercise and fresh air are the methods which are mainly practised, and the much vaunted "short cuts" to sobriety have not succeeded in securing the confidence of the responsible officials. The existing accommodation is on a relatively small scale, more particularly if the statement of one of the Scottish newspapers must be accepted as true that the country has not less than two thousand drunkards who are entitled to be classed as "habitual." The present report will afford those in power some guidance in determining whether institutional treatment should be further extended. Dr. Dunlop strongly urges this policy and draws especial attention to the great want of voluntary retreats for the poorer class and the need for additional inebriate reformatory accommodation.

### On Behalf of the Hospitals.

IT will be remembered that in his speech at Marlborough House on March 8 last, on the occasion of the annual meeting of the General Council of King Edward's Hospital Fund for London, his Royal Highness the Prince of Wales announced that a large sum of money, estimated to produce £4,600 a year, had been promised by a friend, if a further sum of double that amount were obtained by the end of this year. This sum, if forthcoming, will be sufficient to raise the annual income of King Edward's Hospital Fund from investments and other permanent sources to £50,000. With the object of drawing attention to this magnificent offer, and to prevent any chance of the sum offered being lost from failure on the part of the general public to supply the additional two-thirds of the amount required, the Executive Committee of the Fund have issued an appeal, which we publish on another page. We understand that up to the present some £11,500 have been received towards the total asked for, so that a large sum is still necessary, and a magnificent field is open for the exercise of practical philanthropy, as well for those of more modest means who can still give a few pounds, as for the wealthy. Neither should a full response to the appeal now being made be a matter simply of casual inclination; rather it should be looked upon as a duty to give and to thereby offer a token of gratitude and confidence towards an institution which, when our voluntary hospitals were in their direst need, appeared, like the fairy godmother in the play, to effect an entire transformation of their circumstances. All that now remains is to put the finishing touches to the work so well begun, and to place our hospital system for ever beyond the paralysing touch of poverty. It is as true now as it ever was that the wants of the needy might be supplied by the superfluities of the well-to-do. Here, then, is as good an opportunity of acting up to this moral axiom as is ever likely to occur.

